# Screening and identification of host signaling pathways for alleviating influenza-induced production of pro-inflammatory cytokines, IP-10, IL-8, and MCP-1, using a U937 cell-based influenza model

**DOI:** 10.3389/fmicb.2025.1535002

**Published:** 2025-01-27

**Authors:** Si Chen, Yang Yu, Yue Su, Xiaoqin Lian, Lefang Jiang, Zhuogang Li, Mingxin Zhang, Yarou Gao, Haonan Zhang, Xingjian Zhu, Jiaxin Ke, Xulin Chen

**Affiliations:** ^1^Department of Immunology and Microbiology, College of Life Science and Technology, Institute of Medical Microbiology, Jinan University, Guangzhou, China; ^2^State Key Laboratory of Virology, Wuhan Institute of Virology, Chinese Academy of Sciences, Wuhan, China

**Keywords:** influenza virus, pro-inflammatory cytokine, kinase inhibitor, protein tyrosine kinase, mitogen-activated protein kinase, Janus kinase/signal transducers and activators of transcription

## Abstract

Influenza virus infection initiates an exaggerated inflammatory response, which may culminate in a fatal cytokine storm characterized by the excessive production of pro-inflammatory cytokines. Prior research indicates that IP-10, IL-8, and MCP-1, primarily produced by monocytes and macrophages, play a crucial role in influenza-induced inflammation. The lung injury from influenza virus infection can be mitigated by suppressing or inhibiting these cytokines through knockout, knockdown, or targeted intervention approaches. To identify the key host signaling pathways responsible for producing pro-inflammatory cytokines, we utilized a U937 cell model that secretes IP-10, IL-8, and MCP-1 in response to influenza infection. This model has been previously validated in our laboratory as an appropriate system for screening anti-inflammatory agents and potential drug targets. We conducted a screening assay employing an inhibitor library consisting of 2,138 compounds that target various known pathways and host factors. Our findings indicated that inhibitors targeting protein tyrosine kinases and mitogen-activated protein kinases demonstrated superior efficacy in suppressing cytokine production induced by influenza A virus infection compared to inhibitors aimed at other host factors. Notably, a substantial proportion of the identified hits capable of inhibiting the expression of all three cytokines in the secondary screening were classified as tyrosine kinase inhibitors. Validation experiments further established that Janus kinase/signal transducers and activators of transcription (JAK/STAT) pathways, along with p38 MAPK and Raf–MEK–ERK pathways, are the principal regulators of pro-inflammatory cytokine expression in monocytes and macrophages. Moreover, our results suggest that TKIs present promising opportunities as novel therapeutic agents against influenza-induced pneumonia.

## 1 Introduction

The influenza virus infection significantly contributes to morbidity and mortality in the human population. Despite the widespread utilization of antiviral medications in recent years, there has been no substantial reduction in the fatality rate caused by influenza (Klomp et al., 2020). This can primarily be attributed to the lack of effective treatment for severe pneumonia resulting from an infection with this viral pathogen. Research indicates that severe pneumonia is predominantly triggered by an exaggerated immune response of the body toward the influenza virus, leading to excessive production of pro-inflammatory cytokines such as interleukins (ILs), tumor necrosis factor (TNF), interferons (IFNs), and chemokines, collectively referred to as a “cytokine storm” (Gao et al., 2013). These cytokines recruit numerous inflammatory cells that generate excessive chemical components, including reactive oxygen species, which inflict damage upon lung cells, ultimately culminating in acute respiratory distress syndrome.

Although the functions of these cytokines have not been fully elucidated, some are reported to play essential roles in promoting inflammation induced by influenza. For example, compared to the wild-type mice, interferon gamma-induced protein 10 (IP-10) or monocyte chemoattractant protein 1 (MCP-1) gene knockout mice showed more ameliorative inflammation in the lungs, and higher survival rates during influenza virus infection (Lin et al., 2008; Wang et al., 2013). IL-8 receptor (C-X-C chemokine receptor 2, CXCR2) antagonists showed therapeutic effects in influenza-infected mice (Tavares et al., 2017; Washburn et al., 2019). Thus, IP-10, MCP-1, and IL-8 are suggested to be potential targets of anti-inflammatory agents for treating influenza.

Many types of cells (e.g., bronchial and alveolar epithelial cells, endothelial cells, and monocytes/macrophages) produce pro-inflammatory cytokines during influenza virus infection, and different types of cells produce various cytokines. Animal experiments show that influenza virus-induced cytokine production can be divided into two distinct stages. In the first stage, the influenza virus infects epithelial cells to produce a small amount of cytokines and chemokines; in the second stage, these early chemokines recruit innate immune cells (e.g., monocytes, macrophages, and neutrophils) into the lung. These immune cells will produce more pro-inflammatory cytokines through activation by influenza virus or other factors, eventually leading to a “cytokine storm” (Davidson et al., 2014). According to double immunofluorescence staining of lung tissues in fatal influenza-infected cases, monocytes/macrophages are the primary producers of IP-10 and IL-8 (Nakajima et al., 2013). Another study reported that tissue-resident alveolar macrophages secrete MCP-1 to recruit bone marrow-derived monocytes into the lung during acute influenza infection (Rosseau et al., 2000). Several *in vitro* experiments confirmed that monocytes/macrophages can be infected by the influenza virus and produce pro-inflammatory cytokines such as IP-10, MCP-1, and IL-8 (Lee et al., 2017; Sakabe et al., 2011). However, the cellular signaling pathways that regulate the expression of these cytokines in monocytes/macrophages remain unclear.

Multiple monocytic and macrophage-like cell lines, such as monocytic U937 cells, monocytic THP-1 cells, macrophage-like iTHP cells, and iU937 cells (induced by phorbol-12-myristate-13-acetate), were used in the regulation of inflammation (Lamichhane and Puthavathana, 2018; Lund et al., 2016; Mukherjee and Sengupta, 2016). U937 was reported to secret IP-10, MCP-1, and IL-8 among these cell lines during influenza virus infection and showed higher signal-to-background ratios (SBRs) than other cell lines tested (Liu et al., 2019). Recently, we established and validated a U937 cell-based model that supports the replication of different subtypes of influenza viruses and the production of essential pro-inflammatory cytokines (Liu et al., 2019).

This study aims to identify inhibitors that can effectively suppress the production of critical pro-inflammatory cytokines. Additionally, by analyzing the targets and signaling pathways associated with these inhibitors, we aim to find the potential drug targets that regulate the production of these essential pro-inflammatory cytokines. We utilized the U937 cell model to achieve this and screened an inhibitor library of 2,138 compounds. Most of these compounds have known targets covering a wide range of pathways reported in various inflammation-related diseases. Our analysis of all identified hits with inhibitory effects against IP-10, MCP-1, and IL-8 production systematically uncovered the crucial signaling pathways regulating pro-inflammatory cytokine secretion in U937 cells infected by the influenza virus. Notably, many tyrosine kinase inhibitors (TKIs) demonstrated reduced levels for all three cytokines within this cell model. Based on our findings, we propose that TKIs could potentially serve as a new class of anti-inflammatory agents for treating severe influenza.

## 2 Results

### 2.1 PTK and MAPK inhibitors suppress cytokine production induced by influenza A virus infection

To identify compounds capable of inhibiting the production of pro-inflammatory cytokines induced by influenza virus infection, we employed our previously established U937 cell-based influenza model (Liu et al., 2019). This cellular model not only facilitates the screening for inhibitors reducing the levels of IL-8, IP-10, and MCP-1 but also enables the identification of antiviral agents. Our screening library is a customized library ordered from Selleck Chemicals. All compounds are drugs under development, most of which passed clinical trial phase I. The library was consisted of 2,138 inhibitors that target over 200 targets, which can be classified into 22 groups, including angiogenesis, autophagy, cell cycle, cytoskeletal signaling, apoptosis, DNA damage, endocrinology and hormones, epigenetics, G proteins and G protein-coupled receptors (GPCRs), immunology and inflammation, mitogen-activated protein kinase (MAPK), microbiology, metabolism, neuronal signaling, nuclear factor-kappa B (NF-κB), proteases, protein tyrosine kinases, stem cells and Wnt, TGF-β/Smad, transmembrane transporters, and ubiquitin.

**Figure 1:**
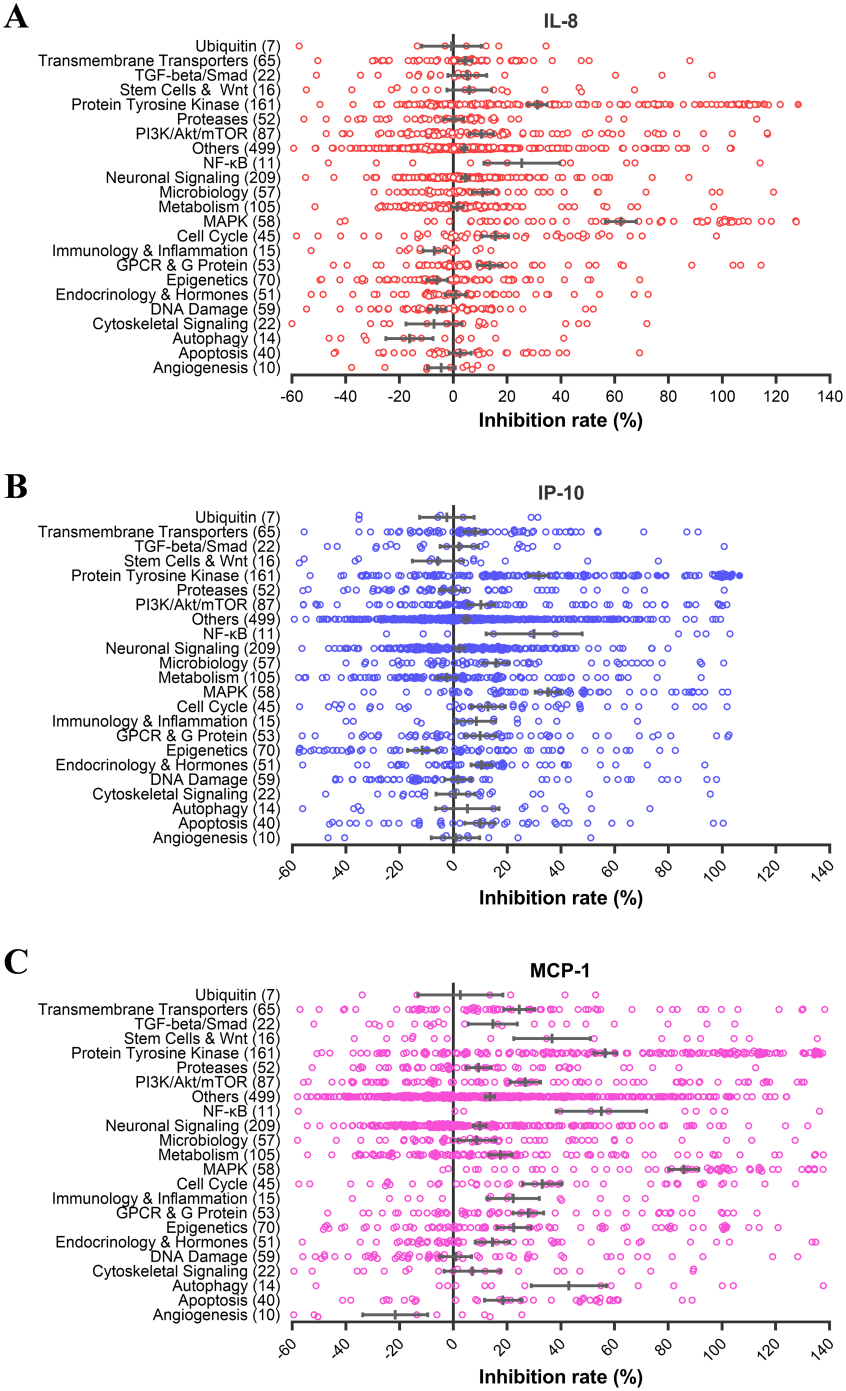
PTK and MAPK inhibitors suppress cytokine production induced by influenza virus infection. U937 cells were infected with influenza A virus A/PuertoRico/8/1934 (MOI = 0.1) with 10 μM of each inhibitor from the compound library and incubated at 37°C for 48 h. The supernatants were harvested, and the concentrations of IL-8, IP-10, and MCP-1 were measured by AlphaLISA. Cell viability was determined using the Cell Titer-Glo reagent, and the inhibition of viral replication was measured using a modified neuraminidase activity assay. Inhibitors with cell viabilities <70% or antiviral activity >80% were excluded to avoid nonspecific anti-inflammatory effects mediated by cytotoxicity or antiviral activity. Inhibition rates of IL-8 **(A)**, IP-10 **(B)**, and MCP-1 **(C)** for each compound from different pathways were shown using hollow circles. The solid circles depict the results of these selected six compounds in Table 1.

In the screen, we measured the protein levels of IL-8, IP-10, and MCP-1 in the culture supernatants of influenza A virus-infected U937 cells treated with 10 μM of each compound and calculated the inhibition rates. The antiviral effect of each compound was tested in parallel. All compounds with antiviral effects (>80%) were excluded for further analysis since reduced virus replication mediates lower-level cytokine response. Notably, the results from a few groups with a limited number of inhibitors, such as NF-κB, were not considered due to their large error bars. Most groups displayed little inhibition (<20% for IL-8 and IP-10, and < 40% for MCP-1), except for the MAPK and protein tyrosine kinase (PTK) groups (Figure 1). The average inhibition by compounds targeting MAPK was 62.3, 35.2, and 85.7% for IL-8, IP-10, and MCP-1 production, respectively. The average inhibition by compounds targeting PTK was 32.9, 33.7, and 57.6% for IL-8, IP-10, and MCP-1 production, respectively. These results indicate that inhibitors from the MAPK and PTK groups have more potential to reduce cytokine production mediated by influenza virus infection than those from other groups. Through further data analysis, we found that the average inhibitory activities of MAPK (61.4%) and PTK (41.4%) groups were significantly higher than those of other groups. This finding suggests that MAPK and PTK signal pathways may play an essential role in the secretion of cytokines in U937 cells upon influenza virus infection.

### 2.2 TKIs account for a large percentage of hits with inhibitory activity on all three cytokines

**Figure 2:**
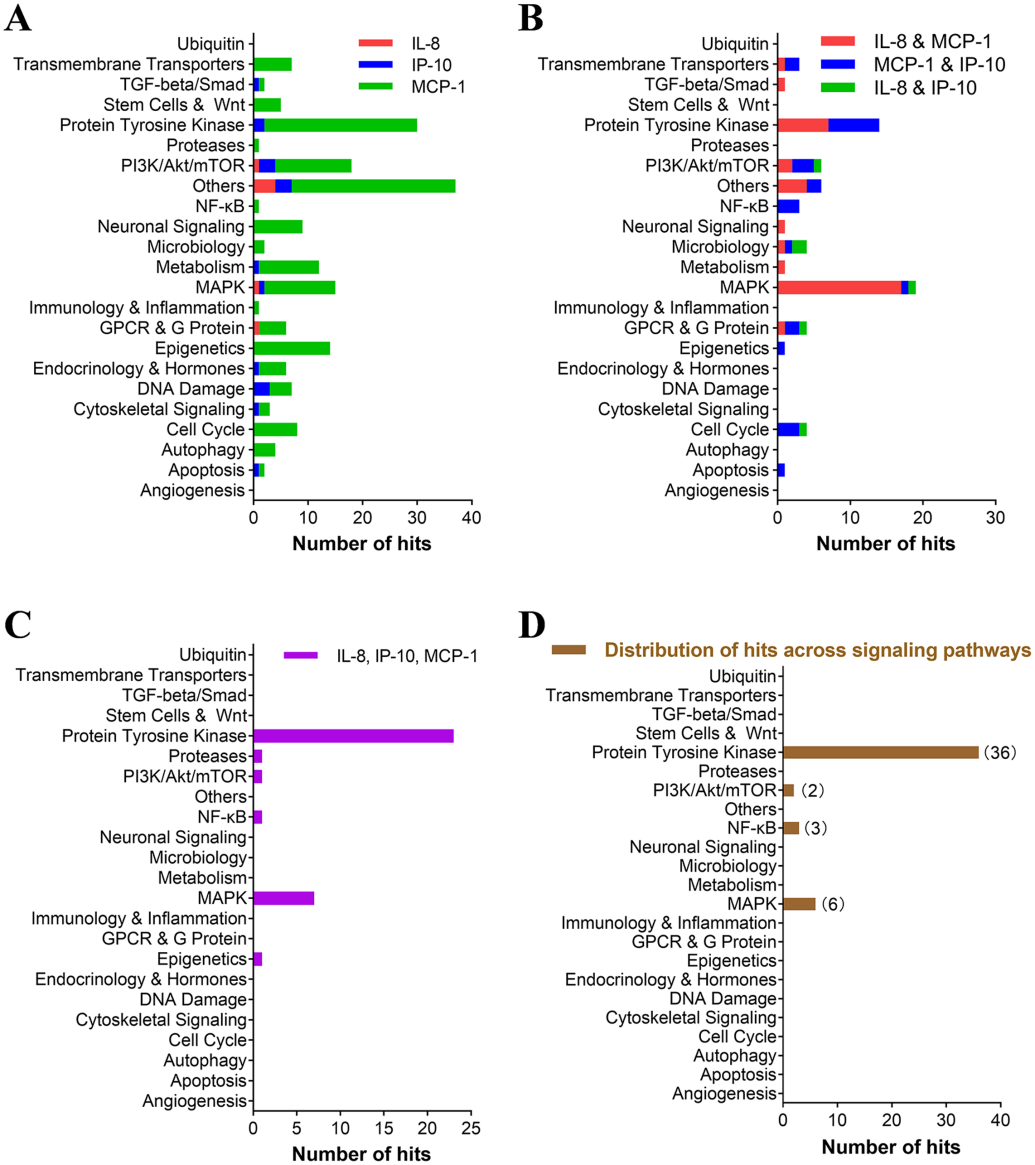
TKIs accounted for a large percentage of hits with an inhibitory effect on all three cytokines. **(A–C)** The pathways targeted by inhibitors with more than 80% inhibition of single cytokines **(A)**, two cytokines **(B)**, and three cytokines **(C)** are represented. Inhibitors that inhibit at least two cytokines **(B,C)** were rescreened in the presence of gradient concentrations of hit compounds. Each selected inhibitor required to reduce cytokine production and cell viability by 50% (IC50 and CC50, respectively) was calculated using Prism v.5 software (GraphPad Software, San Diego, CA). **(D)** The distribution of inhibitors with a selective index (SI) of greater than 10 for inhibiting all three cytokines was further analyzed for their pathway distribution.

Furthermore, highly potent compounds (inhibition rate > 80%) from all categories were selected for a secondary analysis to evaluate their inhibitory effects on the production of the three cytokines. We identified 290 hits capable of inhibiting at least one of the three cytokines. Among these, approximately 200 hits selectively inhibited a single cytokine (primarily MCP-1) with targets spanning various groups including PTK, PI3K/Akt/mTOR, MAPK, and epigenetics (Figure 2A). Sixty-seven compounds exhibited inhibition against two cytokines (mainly IL-8 and MCP-1), predominantly targeting the MAPK group (Figure 2B). Thirty-four compounds demonstrated inhibition against all three cytokines, primarily through targeting the PTK group (Figure 2C). This distribution pattern may be attributed to MAPK inhibitors, particularly MEK inhibitors, showing greater propensity in reducing IL-8 and MCP-1 production while exerting less impact on IP-10 inhibition.

Compounds highly inhibiting the production of two and three cytokines were subjected to the secondary confirmation study on EC50 of multi-cytokine (IL-8, IP-10, and MCP-1) inhibition. Of these compounds, 48 had a selective index over 10 and were identified as “hits” with a hit rate of 2% (48/2138). Notably, 77% (37/48) of hits target PTKs, and 13% (6/48) target the MAPK pathway (p38 and Raf/MEK/ERK). The remaining 10% (5/48) target the PI3K/AKT/mTOR and NF-κB pathways (Supplementary Table 1, Figure 2D).

Next, we investigated the inhibitory effects of hit compounds on the production of IL-8, IP-10, and MCP-1 on U937 cells infected with other influenza viruses, aiming to assess their broad-spectrum activity. Specifically, we selected six compounds for verification on U937 cells infected with human H3N2 and bird H7N8 influenza virus strains. By comparing the selective inhibition of these compounds on cytokine production, we observed that all six compounds exhibited potent inhibitory activity against cytokine induction by both H3N2 and H7N8 viruses, similar to that observed with the H1N1 virus (Table 1).

**Table 1:** Comparison of the anti-inflammatory activities of kinase inhibitors in U937 cells infected with different IAV strains.

Name	CC50 (μM)	EC50 (μM)			SI		
		IL-8	IP-10	MCP-1	IL-8	IP-10	MCP-1
H1N1							
Dasatinib	>100	1.887	1.163	0.190	>53	>86	>526
Ponatinib	10	0.294	0.270	0.385	34	37	26
AZD1480	100	7.692	7.692	6.250	13	13	16
Baricitinib	5.38	0.020	0.020	0.020	>269	>269	>269
R406	>100	0.130	0.431	0.240	>769	>232	>416
GNF-7	13.63	0.019	0.071	0.018	>706	192	769
H3N2							
Dasatinib	>100	0.730	0.017	0.018	137	5,968	5,501
Ponatinib	10	0.714	0.059	0.625	14	169	16
AZD1480	100	7.143	0.017	0.478	14	5,823	209
Baricitinib	5.38	0.215	0.005	0.035	25	1,070	154
R406	>100	0.690	0.005	0.062	>145	>19,607	>1,617
GNF-7	13.63	0.027	0.001	0.014	>500	>20,000	>1,000
H7N8							
Dasatinib	>100	0.135	0.014	0.044	743	7,143	2,277
Ponatinib	10	0.833	0.833	0.345	12	12	29
AZD1480	100	0.215	0.057	0.714	466	1,747	140
Baricitinib	5.38	0.017	0.057	0.053	321	94	101
R406	>100	0.088	0.090	0.035	>1,136	>1,111	>2,857
GNF-7	13.63	0.014	0.005	0.109	>1,000	>2,564	>125

^a^SI, selectivity index is calculated as CC50/EC50.

### 2.3 JAK is an essential target for regulating pro-inflammatory cytokines induced by influenza infection in U937 cells

**Figure 3:**
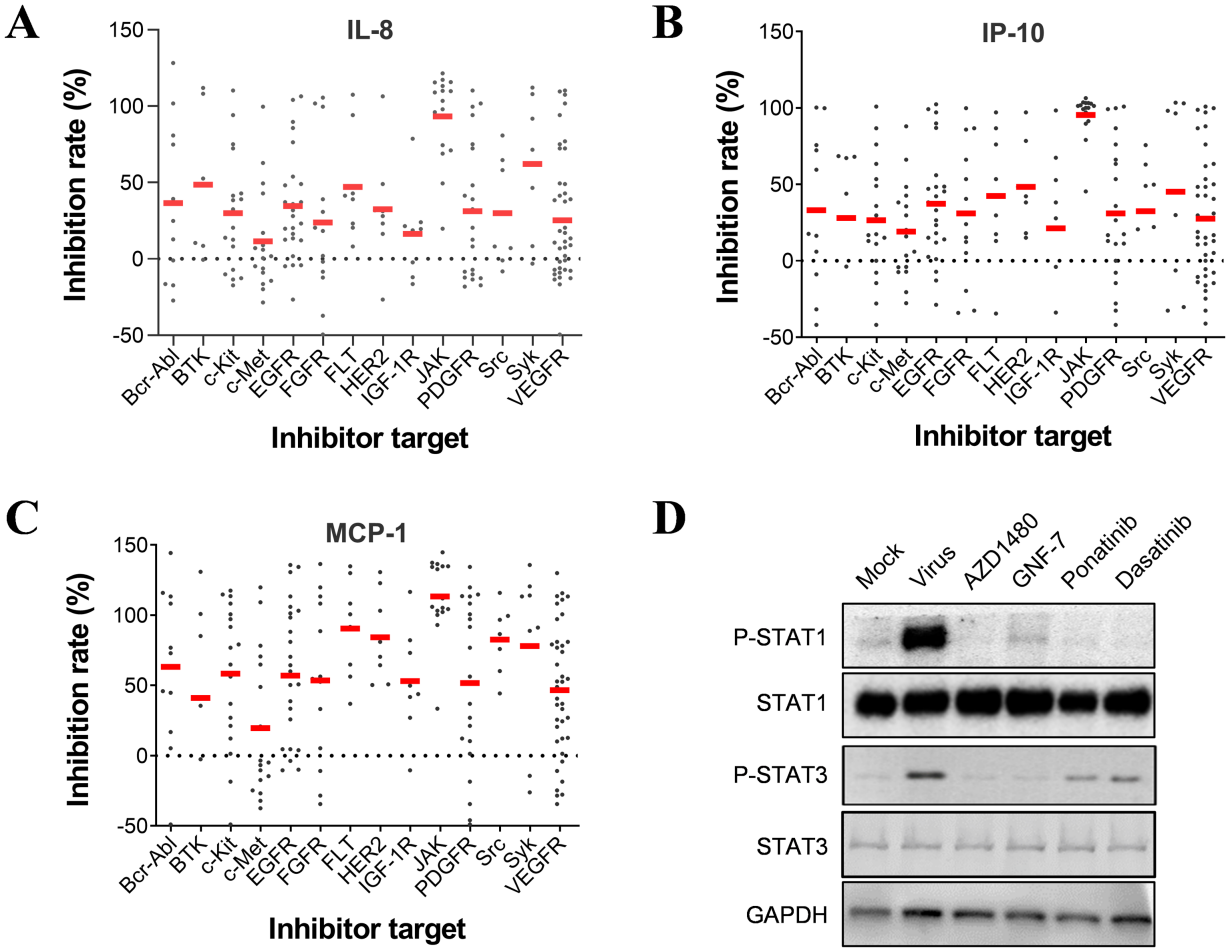
JAK is essential for regulating the production of pro-inflammatory cytokines induced by influenza infection in U937 cells. Inhibition rates on IL-8 **(A)**, IP-10 **(B)**, and MCP-1 **(C)** by each compound in the PTK group are categorized based on targets. **(D)** U937 cells were infected with influenza A/PuertoRico/8/1934 (MOI = 0.1) and treated with 50 μM AZD1480, 1 μM GNF-7, 1 μM ponatinib, or 1 μM dasatinib (all at maximum non-toxic concentration) respectively. The cells were then lysed after 24 h incubation at 37°C, and the levels of phosphorylated STAT1 (p-STAT1), total STAT1, phosphorylated STAT3 (p-STAT3), and total STAT3 were assessed by western blot.

The results from the secondary screen indicate that many TKIs exhibit high anti-inflammatory activities against all three cytokines. Analysis of the subgroups within the PTK group revealed that JAK kinase inhibitors accounted for a significant proportion and displayed inhibitory effects on all three pro-inflammatory cytokines (Figures 3A–C). Further examination of highly active inhibitors targeting other pathways indicated that several multi-target inhibitors, such as Ponatinib and Dasatinib, may also act on JAK kinases. To investigate whether influenza virus infection activates the JAK–STAT pathway, we conducted western blot assays, which confirmed its activation and subsequent increase in STAT1 phosphorylation levels as a downstream signaling molecule. Interestingly, PTK inhibitors, including Ponatinib, Dasatinib, and GNF-7, significantly reduced STAT1 phosphorylation levels similar to JAK inhibitor AZD1480 despite their original targets not being JAK kinases (Figure 3D). Therefore, it can be inferred that JAK serves as an essential regulatory site for inflammatory factors in influenza virus-infected U937 cells.

### 2.4 The JAK-STAT signal pathway is crucial in regulating IP-10 expression on U937 cells

**Figure 4:**
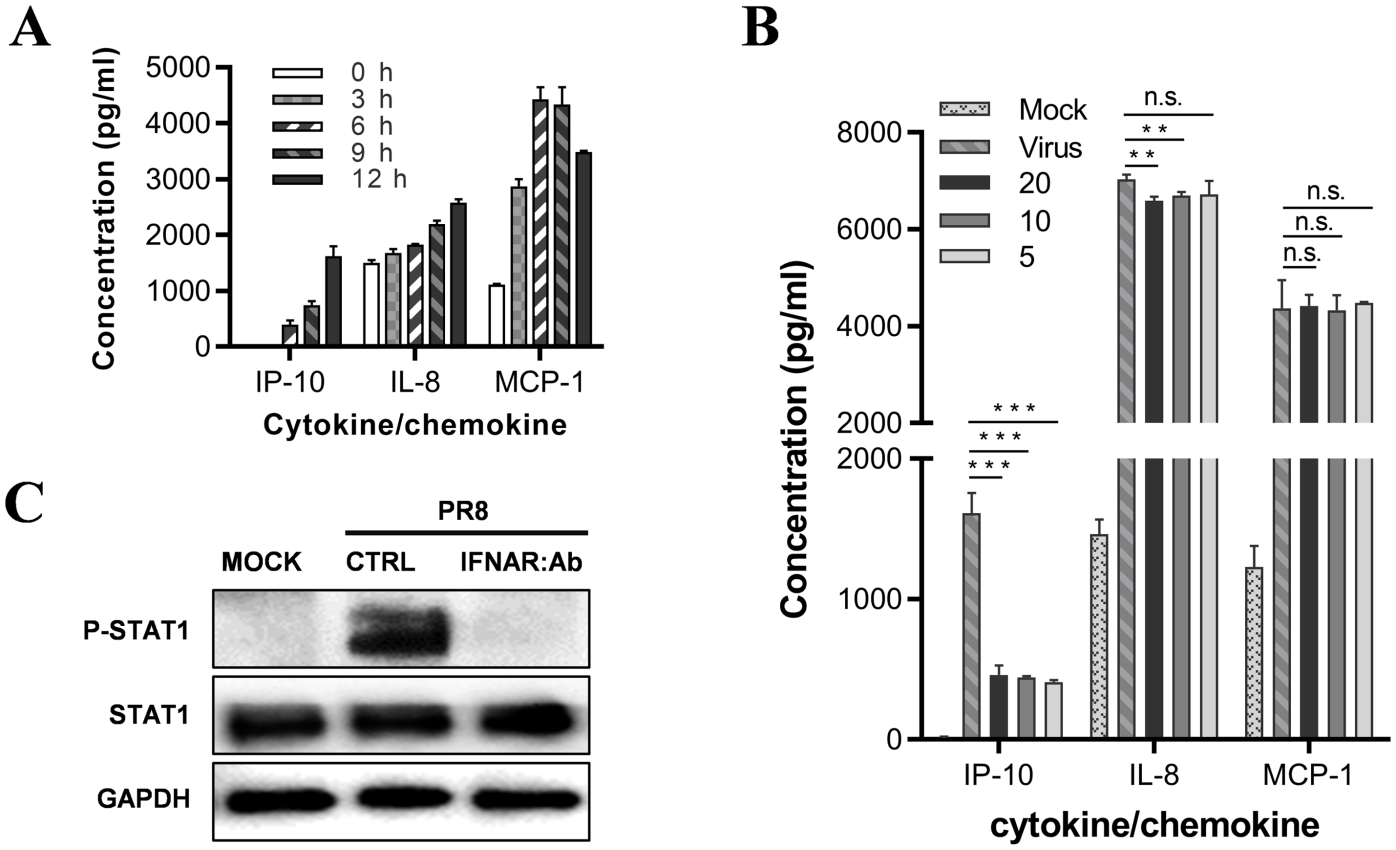
JAK–STAT is the signal pathway regulating IP-10 expression on U937 cells. **(A)** U937 cells were cultured in the presence of 500 U of IFN-α2b. The supernatants were harvested at different time points, and the levels of IL-8, MCP-1, and IP-10 were measured by AlphaLISA. **(B)** U937 cells were infected with influenza A/PuertoRico/8/1934 virus (MOI = 0.1) and treated with different concentrations of antibody against the type I IFN receptor (clone: MMHAR-2, PBL Assay Science) and then incubated at 37°C. The supernatants were harvested 48 h post-infection, and levels of the IL-8, MCP-1, and IP-10 were measured by AlphaLISA. **(C)** The cells were lysed 24 h post-infection, and the levels of phosphorylated STAT1 (p-STAT1) and total STAT1 were assessed by western blot. The data are representative of at least three independent experiments. **p < 0.01; ***p < 0.001. n.s., not significant.

The JAK-STAT signal pathway is responsible for the downstream effects of interferon receptors. Studies have shown that, after an influenza virus infection, type I interferon is secreted by U937 cells (Lamichhane et al., 2018). To investigate whether type I interferon can regulate IP-10, IL-8, and MCP-1 levels, we treated U937 cells with IFN-a. We observed a significant up-regulation in the secretion of IP-10, IL-8, and MCP-1 upon IFN-a treatment (Figure 4A). Based on this finding, we assume that type I interferons may play a critical role in regulating the secretion of pro-inflammatory cytokines. To test this hypothesis, we infected U937 cells with the influenza virus and treated them with a neutralizing antibody for the type I interferon receptor. We found that IP-10 secretion was significantly inhibited by as low as 5 mg/mL of neutralizing antibody. At the same time, the other two inflammatory factors were virtually unaffected, even at as high as 20 mg/mL of the neutralizing antibody (Figure 4B).

We conducted a western blot assay to confirm that influenza virus infection of U937 cells activates the JAK-STAT pathway through IFN-a; the results showed that STAT1 is phosphorylated upon influenza virus infection, suggesting that the JAK-STAT pathway was activated. Furthermore, treatment with 5 mg/mL neutralizing antibody against the receptor of IFN-a completely abolished the phosphorylation of STAT1, suggesting that the JAK-STAT pathway is activated through type-I interferon (Figure 4C).

Therefore, our findings suggest that the JAK-STAT pathway regulates IP-10 expression in U937 cells during influenza virus infection, while alternative signaling pathways are likely involved in regulating IL-8 and MCP-1 levels.

### 2.5 Raf/MEK/ERK and p38 signal pathways regulate cytokines production on U937

**Figure 5:**
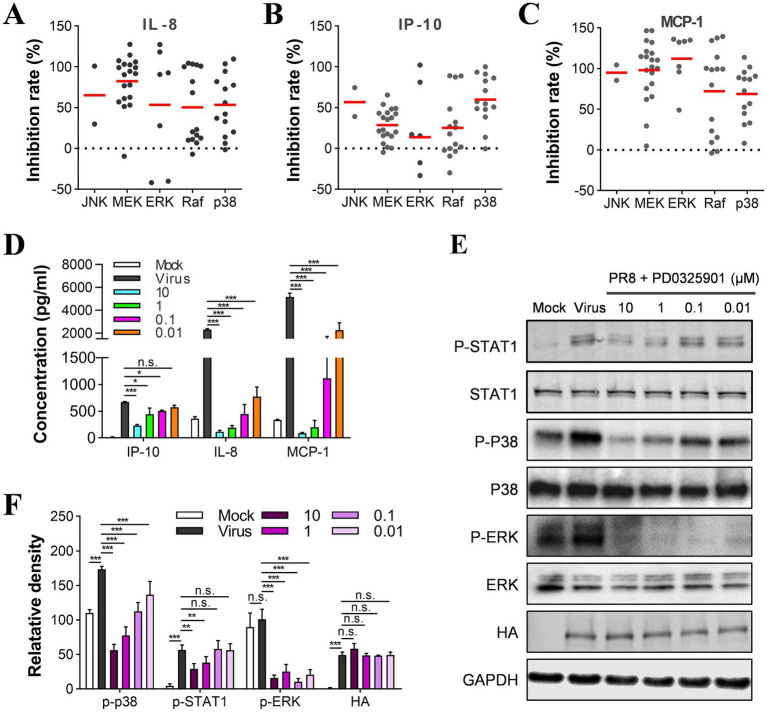
Raf/MEK/ERK and p38 signal pathways are involved in the regulation of cytokines in U937 cells. **(A–C)** Determination of inhibition rates on IL-8, IP-10, and MCP-1 by each compound in the MAPK group and the distribution of targets between subgroups. U937 cells were infected with influenza A/PuertoRico/8/1934 virus (MOI = 0.1) in the presence of 10 μM of each inhibitor and then incubated at 37°C for 48 h. The distribution of the targets of all the inhibitors in the MAPK group was then analyzed and categorized into subgroups. **(D)** U937 cells were infected with influenza A/PuertoRico/8/1934 (MOI = 0.1) in the presence of different concentrations of PD0325901 and then incubated at 37°C. The supernatants were harvested 48 h post-infection, and the levels of IL-8, MCP-1, and IP-10 were measured by AlphaLISA. **(E)** The cells were treated as in **(C)** except being lysed 24 h post-infection, and the levels of phosphorylated STAT1, p38, ERK (p-STAT1, p-p38, p-ERK), total STAT1, p38, ERK, and viral protein HA were assessed by western blot. **(F)** A quantitative analysis of the relative levels of the phosphorylated STAT1, p38, ERK (p-STAT1, p-p38, p-ERK) in **(E)**. The data are representative of at least three independent experiments. n.s. no significance; *p < 0.05; **p < 0.01; ***p < 0.001.

In our primary screen (Figure 2B), we observed that many MAPK inhibitors suppressed the production of both IL-8 and MCP-1. To analyze the distribution of the targets of these inhibitors in the MAPK group, we investigated the inhibition of IL-8 and MCP-1 by inhibitors of Raf, MEK, and ERK, which are the upstream and downstream signaling molecules of the MAPK pathway. Our analysis showed that MEK inhibitors had the highest inhibitory effect on IL-8 and MCP-1, while inhibitors of Raf and ERK also tended to inhibit these molecules (Figures 5A–C). Therefore, these results suggest that the Raf/MEK/ERK signaling pathway regulates IL-8 and MCP-1. We used MEK inhibitor PD0325901 as the reference compound to test this hypothesis. We found that despite the phosphorylation level of ERK not increasing significantly upon influenza virus infection, PD0325901 inhibited the secretion of IL-8 and MCP-1 (Figure 5D) and decreased the phosphorylation level of ERK (Figure 5E). At high concentrations, PD0325901 was also found to inhibit the secretion of IP-10 and the phosphorylation of STAT1 proteins (Figures 5D,E).

We analyzed the anti-inflammatory activity of inhibitors of JNK and p38, which also belong to the MAPK family. We found that the p38 inhibitors showed slightly less anti-inflammatory activity on the secretion of IL-8 and MCP-1 than the MEK and ERK inhibitors (Figures 5A–C). According to the western blot results, the phosphorylation level of p38 was reduced upon treatment with the MEK inhibitor (Figures 5E,F). Thus, the p38 pathway was also suggested to regulate the cytokine response mediated by influenza virus infection. It is worth noting that the number of JNK inhibitors is limited in this library. Most potent inhibitors for producing IL-8, IP-10, and MCP-1 showed potent cytotoxicity on U937 cells; therefore, our study could not evaluate their anti-inflammatory activity.

### 2.6 TKIs repress cytokine production by suppressing the p38 pathways

**Figure 6:**
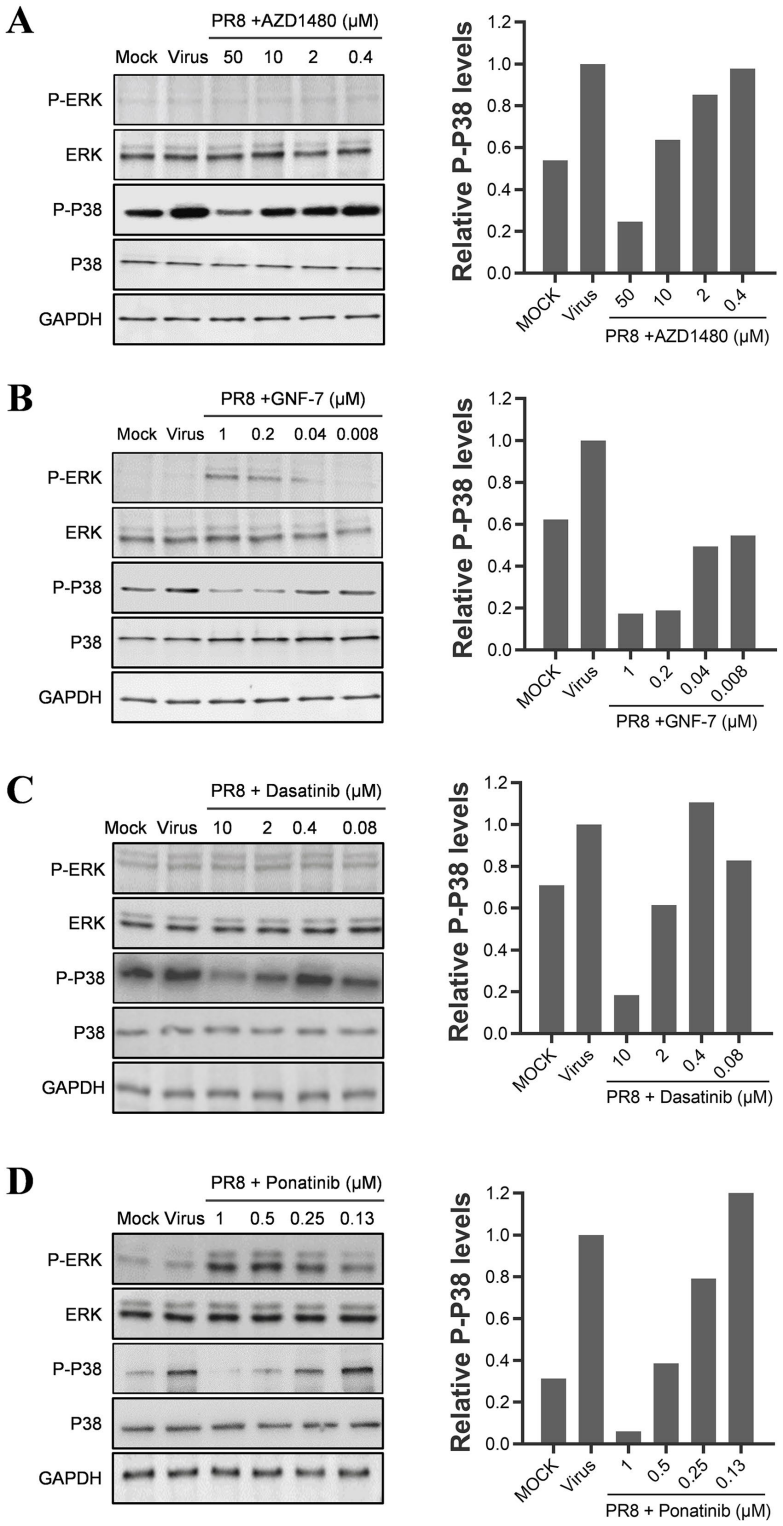
TKIs repress cytokine production by suppressing the p38 pathway. U937 cells were infected with 0.1 MOI of influenza A/PuertoRico/8/1934 virus in the presence of different concentrations of AZD1480 **(A)**, GNF-7 **(B)**, Dasatinib **(C)**, and Ponatinib **(D)**, then incubated at 37°C. Cells were lysed 24 h post-infection, and the levels of phosphorylated p38 and ERK (p-p38 and p-ERK) and total p38 and ERK were assessed by western blot. A quantification of phosphorylated p38 levels for each drug treatment was included in the right panel of each figure.

As previously mentioned, TKIs have been found to inhibit the JAK–STAT pathway, which regulates the production of only IP-10 among the three cytokines we studied. We hypothesize that TKIs might also be able to inhibit the MAPK pathways. To test this hypothesis, we performed a western blot analysis to examine the effects of viral infection and TKI treatment on the phosphorylation levels of ERK and p38. Our results showed that the phosphorylation of p38 increased during the influenza A virus infection. Also, we noticed that the mock-infected cells consistently showed a basal level of p38 phosphorylation. We suspect that the p38 pathway may have been activated by an unknown stimulus during the experimental procedures. However, the four TKIs we tested reduced the phosphorylation of p38 in a concentration-dependent manner, regardless of whether it is activated by influenza virus infection or another unknown stimulation (as shown in Figures 6A–D). On the other hand, the phosphorylated levels of ERK in U937 cells did not change significantly upon influenza A virus infection. Interestingly, two TKIs, GNF-7 and ponatinib, promoted ERK phosphorylation despite suppressing cytokine production. These findings suggest that ERK may not be the critical regulator of the production of the three cytokines (as shown in Figures 6B,D).

### 2.7 TKIs targeting JAK and MEK pathways display off-target effects on closely related kinases

Results from Western blot analysis revealed that tyrosine kinase inhibitors (TKIs) effectively target the catalytic domains of protein kinases, thereby inhibiting the JAK–STAT and p38 pathways (Figures 3, 6). These domains exhibit a conserved Asp-Phe-Gly (DFG) motif within the ATP-binding cleft of tyrosine kinases SRC, ABL, and c-KIT, as well as in the serine/threonine kinase p38α (Karaman et al., 2008). Certain TKIs, such as ponatinib and dasatinib, specifically interact with this motif in select kinases (Ranjitkar et al., 2010). However, due to their limited selectivity among different types of tyrosine kinases, other classes of TKIs like ABL and Syk inhibitors may also impact the JAK–STAT pathway (Choi et al., 2010; Blunt et al., 2017).

We selected several highly potent TKI compounds and evaluated their inhibitory activities against kinases using cell-free kinase assays. Subsequently, we compared these activities with their effects on cytokine production in the U937 cell-based model. Our findings indicate that the inhibitory activities of these compounds (represented by EC50 values) against kinases *in vitro* are generally higher than those against cytokines in the U937 cell model, possibly due to factors such as drug absorption (Tables 1, 2). Furthermore, our investigation revealed that Ponatinib, a multi-target inhibitor, can directly inhibit JAK1 activity. This confirms our previous hypothesis regarding the off-target effects of non-JAK TKIs interfering with JAK signaling pathways. Interestingly, all four tested JAK inhibitors, Tofacitinib, Decernotinib, Baricitinib, and AZD1480, inhibited both JAK1 and JAK2 regardless of their initial intended targets. In contrast to other inhibitors examined here, Ponatinib primarily targeted JAK1, which had a weaker effect on JAK2 (Table 2). Consequently, it displayed limited inhibition of STAT3 phosphorylation (Figure 3D). Additionally, we observed that inhibitors targeting both JAK and MEK pathways, including Baricitinib, AZD1480, and PD0325901, failed to inhibit p38 kinase activity *in vitro* (Table 3). However, western blot experiments demonstrated their ability to suppress p38 phosphorylation in cellular models (Figures 5E, 6; Table 4).

**Table 2:** Comparative analysis of the inhibitory activities of JAK Inhibitors on JAK1 and JAK2 kinases *in vitro*, and the production of IP-10 in U937 Cells infected with influenza virus.

Name	Target	IC50 (μM)		
		JAK1	JAK2	IP-10
Tofacitinib	JAK3	0.0014	0.005	0.02
Decernotinib	JAK3	0.013	0.09	0.1
Baricitinib	JAK1/2	0.001	0.001	0.02
AZD1480	JAK2	0.43	<0.2	7.11
Ponatinib	ABL	0.21	>1	0.25

**Table 3:** Comparison of the inhibitory activities of kinase inhibitors against p38 kinases *in vitro* and the production of IL-8 and MCP-1 in U937 cells infected with influenza virus.

Name	Target	IC50 (μM)		
		p38	IL-8	MCP-1
BIRB 796	p38	0.6	5.2	10
LY2228820	p38	0.06	0.005	2.2
PD0325901	MEK	>1	<0.02	<0.02
Baricitinib	JAK1/2	>1	<0.02	<0.02
AZD1480	JAK2	>100	7.5	6.3

**Table 4:** Comparison of the inhibitory activities of kinase inhibitors on the phosphorylation of p38 *in vitro* and the production of IL-8 and MCP-1 in U937 cells infected with influenza virus.

Name	Target	IC50 (μM)		
		p-p38	IL-8	MCP-1
Dasatinib	c-KIT	1.0	1.90	0.19
Ponatinib	ABL	0.18	0.30	0.40
PD0325901	MEK	0.01	<0.01	0.01
GNF-7	ABL	0.01	<0.02	<0.02
AZD1480	JAK2	5.3	7.5	6.3

These results suggest that many TKIs targeting JAK and MEK pathways have off-target effects on closely related kinases, leading to broader inhibition of cytokine production and potential toxicity to host cells.

## 3 Discussion

The therapeutic efficacy of current direct-acting antiviral (DAA) drugs in treating influenza is limited. These drugs typically require administration within 48 h after symptom onset to achieve optimal therapeutic outcomes. Failure to meet this time window may result in high viral titers, leading to excessive inflammation and severe manifestations such as acute respiratory distress syndrome and even fatality. Traditional anti-inflammatory agents like glucocorticoids exhibit ineffectiveness against influenza-induced inflammation. Studies propose that specific targeting pro-inflammatory cytokines produced abundantly, including IL-8, IP-10, and MCP-1, with anti-inflammatory medications holds promise for mitigating influenza-associated inflammation. However, only a few inhibitors of pro-inflammatory cytokines have been reported thus far for their ability to suppress influenza-induced inflammatory responses.

This study utilized the monocytic cell influenza infection model, known as U937, which was recently established and validated in our laboratory. When infected with the influenza virus, these cells produce cytokines such as IL-8, IP-10, and MCP-1, which are responsible for the pathogenesis of influenza (Liu et al., 2019). We conducted a high-throughput drug screening of anti-inflammatory agents to identify inhibitors of the signaling pathways and molecules that produce these cytokines. The results showed that PTK and MAPK inhibitors had the most significant effects on cytokine production, while NF-κB and PI3K/Akt/mTOR inhibitors had a lesser impact. Although some studies have suggested that NF-κB and PI3K inhibitors display anti-inflammatory activity during influenza infection, further investigation is necessary to understand the roles of NF-κB and PI3K pathways in cytokine expression regulation on the U937 model (Lamichhane et al., 2018; Lu et al., 2011; Ehrhardt et al., 2013).

Notably, our study omitted the consideration of stimulatory effects for those inhibitors with negative inhibition rates well below 0 %, which may arise from the activation of the inflammatory response, suggesting the necessity for further experimental validation. The exploration of compounds demonstrating stimulatory effects on influenza-induced inflammation bears significance and implications for averting the utilization of such agents during influenza virus infection.

In our immunomodulatory drug screening against influenza-induced inflammation, most JAK inhibitors showed high anti-inflammatory activity. Janus (JAK) kinases are a kind of non-receptor tyrosine kinase that was activated by cytokine (e.g., IFN) receptor signaling, followed by interacting with signal transducers and activators of transcription (STAT) proteins to make the latter phosphorylated (Schindler et al., 2007). Ohmori et al. reported that the phosphorylated STATs form dimers and enter the nucleus to bind to the ISRE motifs in the promoter of the IP-10 gene (Ohmori and Hamilton, 1993). Our study found that IFNAR-neutralizing antibodies could significantly reduce the production of IP-10 on U937 cells infected by the influenza virus. Based on the report that U937 cells produce type I IFN after infection with influenza viruses, we propose that the JAK–STAT pathway capable of activation by type I IFNs was the primary regulator of IP-10 production on U937 cells.

Our screening results have indicated that the MAPK pathway plays a role in the production of cytokines induced by influenza. This pathway encompasses three subfamilies: ERK, p38, and JNK. TLR3 activates MAPKs by sensing dsRNA generated during influenza virus replication and targeting downstream transcription factors such as Activator Protein 1 (AP-1) and CCAAT/enhancer-binding protein (C/EBP) (Yamamoto et al., 2003; Esteves et al., 2013; Mishra et al., 2014). According to point mutation analysis, the promoter region of genes that encode cytokine IL-8 and MCP-1 includes AP-1 and C/EBP elements critical for cytokine expression (Peng et al., 2016; Yan et al., 2019). Our study has shown that inhibiting Raf/MEK/ERK can significantly decrease cytokine production, particularly IL-8 and MCP-1. Reports from others have shown that the same regulation can be observed in the A549 cell model. Additionally, some MEK inhibitors have been reported to protect mice infected with a lethal dose of influenza virus (Schraeder et al., 2018; Pinto et al., 2011). However, in this study, we did not observe any antiviral activity of MEK inhibitors in U937 cells infected with the influenza virus.

We performed two rounds of screening to exclude compounds that exhibited toxicity-mediated inhibition. Our results revealed that tyrosine kinase inhibitors (TKIs) constituted a significant proportion of compounds with potent inhibitory activity against all three cytokines. TKIs are commonly employed in targeted cancer therapy, and their potential application in anti-inflammatory treatment is being actively explored. Certain JAK inhibitors, such as Baricitinib and Tofacitinib, have been clinically used to treat inflammatory diseases like rheumatoid arthritis and systemic lupus erythematosus (Norman, 2014). SYK inhibitors have demonstrated anti-inflammatory effects in *Pseudomonas aeruginosa* infection models (Aval et al., 2013; Alhazmi et al., 2018). Notably, recent studies by ourselves and others have reported that two TKIs, namely ibrutinib and ponatinib, exhibit broad-spectrum inhibition of cytokine storm in the lungs, thereby protecting mice from lethal influenza infection without compromising viral titers (Florence et al., 2018; Chen et al., 2019). By integrating our findings with other observational data, we propose that TKIs hold promise for potential applications in influenza treatment.

In the context of influenza virus infection, the combined activities of lung epithelial cells, neutrophils, and monocytes/macrophages contribute to the observed cytokine storm *in vivo*. It is our assertion that no single cell type can fully represent the diverse inflammatory responses to influenza virus infection *in vivo*. Nevertheless, certain pathways and regulatory mechanisms governing cytokine production during influenza virus infection may be shared or similar between U937 cells and the lungs of mice. Consequently, we argue that the production of the three specified cytokines by infected U937 cells is biologically relevant to the multifaceted *in vivo* cytokine storm mechanism, albeit not in quantity. However, the relevance of inhibiting the three selected cytokines in influenza virus-infected U937 cells to suppressing the cytokine storm *in vivo* is uncertain, and further *in vivo* studies are necessary to validate the efficacy of targeting the identified kinases, particularly PTKs.

**Figure 7:**
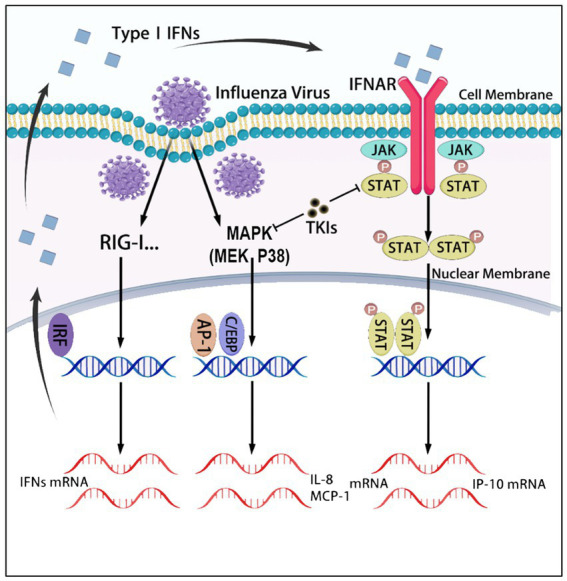
The graphical abstract summarizes the signaling pathways regulating the expression of IL-8, MCP-1, and IP-10, which are targeted by tyrosine kinase inhibitors (TKIs). In U937 cells, influenza virus infection activates the p38 MAPK signaling pathways, leading to the production of IL-8 and MCP-1. Additionally, the virus infection induces the production of interferons, which activate the JAK–STAT pathway and promote the expression of IP-10. TKIs inhibit the production of these three cytokines by blocking the p38 and JAK–STAT signaling pathways.

In conclusion, this study has identified many new anti-inflammatory agents, their target molecules, and pathways that play a crucial role in the influenza-induced cytokine storm, which occurs in a monocyte influenza virus infection model. The JAK–STAT, Raf/MEK/ERK, and p38 pathways are significant cytokine regulators on influenza virus-infected monocytic cells. It has been found that TKIs are a significant class of inhibitors that can reduce all three cytokine productions in influenza virus-infected monocytic cells through JAK–STAT and p38 pathways (as shown in Figure 7). Further efficacy studies are necessary to evaluate the therapeutic effects of inhibitors of the screened-out pathways in the treatment of influenza in the animal model. Notably, we recently demonstrated that a panel of JAK inhibitors effectively lowers the inflammatory response and protects mice from lethal influenza virus infection (Yu et al., 2024). This suggests that JAKs are promising anti-inflammatory drug targets. We expect that further studies may lead to identifying novel valid immunomodulatory targets (pathways) for the anti-inflammatory therapy of influenza pneumonia. Many TKIs have been proven in the clinic for anti-tumor therapy, and their pharmacokinetic and adverse effects have been thoroughly investigated. Unlike long-term cancer treatments, anti-influenza therapies are short-course, which minimizes adverse effects (Planz, 2013). Consequently, TKIs hold immense potential as a new class of immunomodulators for severe influenza treatment.

## 4 Materials and methods

### 4.1 Cell lines and virus strains

Human monocytic cell line U937 (ATCC CRL-1593.2) was maintained in RPMI-1640 medium at 37°C in a 5% CO_2_ incubator. All media were supplemented with 10% fetal bovine serum, 100 U/mL penicillin, and 100 U/mL streptomycin. Influenza virus strain A/PuertoRico/8/1934 (H1N1), A/Human/Hubei/3/2005 (H3N2), and A/Duck/Hubei/216/1983 (H7N8) were provided by the virus collection at the Wuhan Institute of Virology of the Chinese Academy of Sciences in China. Virus stocks were prepared using 10-day-old embryonated chicken eggs. The virus titers were measured using a 50% tissue culture infective dose (TCID_50_) assay in MDCK cells. All experiments in this study were conducted in the biosafety level 2 laboratory.

### 4.2 Chemicals and drug library

The compounds applied in the screen library were purchased from Selleck Chemicals (Shanghai, China). This customized library included drugs in development, with most having passed Phase I clinical trials and with known targets. The library consisted of 2,138 bioactive compounds with a > 95% purity. All compounds were dissolved in DMSO as a 10 mM stock solution. Ponatinib, Dasatinib, GNF-7, Baricitinib, R406, and PD0325901 were purchased from MedChemExpress Co., Ltd. (Shanghai, China). All test compounds were initially dissolved in DMSO.

### 4.3 High-throughput screening of inhibitor library

In the primary screen, 2,138 compounds from the inhibitor library were added individually to 384-well source plates (Labcyte, LP-0200) with 320 compounds per plate. Subsequently, 80 nL of each inhibitor compound, reference compound (Ribavirin), or DMSO were transferred to sterile, clear-bottom ViewPlate 384-well plates (PerkinElmer, 6007460) using an acoustic droplet ejection (ADE) system (Echo 550, Labcyte, CA, United States). Eighty μL medium, which contains U937 cells at a density of 1 × 10^6^ cells/mL infected with 0.05 multiplicity of infection (MOI) of the A/PuertoRico/8/1934 (H1N1) virus, was added to each well to dilute each compound to a final concentration of 10 μM. Wells containing uninfected cells serve as the negative control. After incubating at 37°C for 48 h, the supernatants were taken to detect NA activity and cytokine levels. The remaining cells were tested for cell viability.

The inhibition rate for antiviral activity is defined as “100% minus the percentage of virus production in the drug-treated group compared to that of the virus control group.” The inhibition rate for anti-inflammatory activity is defined as “100% minus the percentage of cytokine/chemokine production in the drug-treated group compared to that of the virus control group.” Compounds that showed more than 80% inhibition and less than 70% cytotoxicity from the primary screen were subjected to a secondary screen. We tested the kinetic effects of these compounds on cytokine inhibition and cytotoxicity over a range of concentrations from no impact to a maximum effect using cytokine inhibition and cytotoxicity assays, as described below.

### 4.4 Neuraminidase activity assay

Supernatants were transferred to 96-black-well plates and incubated at 37°C for 1 h with 20 μM of 2-(4-methylumbelliferyl)-a-d-N-acetylneuraminic acid sodium salt (Sigma, cat. no. M8639) dissolved in 33 mM 2-(N-morpholino) ethane sulfonic acid (pH 6.5) and 4 mM CaCl_2_. The fluorescence intensity was measured at excitation and emission wavelengths of 355 and 485 nm using a multi-label plate reader (Wallac Envision, PerkinElmer, MA, United States).

### 4.5 Alphalisa assay

The Alphalisa kits for detecting levels of IL-8 (AL224C), MCP-1 (AL244C), and IP-10 (AL259C) were purchased from PerkinElmer (PerkinElmer, MA, USA). The Alphalisa assay was performed according to the manufacturer’s protocol. In summary, 20 μl of acceptor beads and 5 μL of supernatant were added to each well of 384-well OptiPlates. The plates were then incubated in the dark at room temperature for 1 h. Next, 25 μL of donor beads coated with streptavidin, which captures the biotinylated antibody, was added. After the assay plates were incubated in the dark at room temperature for another 0.5 h, they were read in Alphalisa mode on an Envision plate reader (Wallac Envision, PerkinElmer, MA, United States).

### 4.6 Cell viability assay

Cell viability was determined by CellTiter-Glo Luminescent Viability Assay according to the provided protocol (Promega). After adding 60 μL of CellTiter-Glo reagent, plates were incubated at room temperature for 15 min with shaking. The luminescence intensity in each well was determined using a multi-label plate reader (Wallac Envision, PerkinElmer, MA, United States). Cell viability is defined as “the percentage of cell viability in the drug-treated cells compared to that of the untreated cells.

### 4.7 Western blot assay

Cells were homogenized in RIPA lysis buffer containing a 1% protease inhibitor cocktail (Roche) to extract total protein. Equal amounts of protein homogenates were separated by SDS-PAGE (Bio-Rad) and transferred onto polyvinylidene difluoride membranes (pore size 0.45 μM; Bio-Rad). The membranes were then blotted with monoclonal antibodies against GAPDH (1:1000, ZSGB-BIO), p38, phosphor-p38, ERK, phospho-ERK, STAT1, phosphor-STAT1 (1:1000; Cell Signaling Technology), STAT3, and phosphor-STAT3 (1:1000; Cell Signaling Technology), respectively. Protein levels were detected with horseradish peroxidase-tagged secondary antibodies and enhanced chemiluminescence western blot reagents (Advansta), followed by visualization using an enhanced chemiluminescence system (AlphaEase FluorChem System, Alpha Innotech Corp.). Bands were quantified using ImageJ (Ver. 1.46; NIH, Bethesda, MD).

### 4.8 Cell-free kinase activity assay

The sources of JAK1 and JAK2 were provided by KinaseProfiler™ (Eurofin, Brussels, Belgium). JAK1 was incubated with 20 mM Tris/HCl pH 7.5, 0.2 mM EDTA, 500 μM GEEPLYWSFPAKKK, 10 mM Magnesium acetate, and [*γ*- 33P]-ATP (specific activity and concentration as required). JAK2 was incubated with 8 mM MOPS pH 7.0, 0.2 mM EDTA, 100 μM KTFCGTPEYLAPEVRREPRILSEEEQEM FRDFDYIADWC, 10 mM Magnesium acetate and [γ-33P]-ATP (specific activity and concentration as required). SRPK2 was incubated with 8 mM MOPS pH 7.0, 0.2 mM EDTA, 300 μM RSRSRSRSRSRSRSR, 10 mM Magnesium acetate, and [γ-33P]-ATP (specific activity and concentration as required). Compounds were added to the assay wells, and reactions were initiated by adding the Mg/ATP mix. After incubation for 40 min at room temperature, reactions were stopped by adding phosphoric acid to a concentration of 0.5%. Ten μL of the response was then spotted onto a P30 filter mat and washed four times for 4 min in 0.425% phosphoric acid and once in methanol before drying and scintillation counting.

### 4.9 Statistical analysis

The concentrations required to inhibit cytokine production by 50% (EC50), reduce cell viability by 50% (CC50), and selective indices (SIs, which are equal to CC50/EC50) of compounds were calculated using Prism v. five software (GraphPad Software, San Diego, CA). Data were presented as mean ± SD for each point. Differences between averages between control samples and tests were statistically analyzed using Student’s t-test; *p*-values <0.05 were considered statistically significant.

## Data Availability

The original contributions presented in the study are included in the article/Supplementary material, further inquiries can be directed to the corresponding author.
