# Supplementary material for: Screening and identification of host signaling pathways for alleviating influenza-induced production of pro-inflammatory cytokines, IP-10, IL-8, and MCP-1, using a U937 cell-based influenza model
Source: Front Microbiol. 2025 Jan 27;16:1535002. doi: 10.3389/fmicb.2025.1535002 (PMC11808136; doi:10.3389/fmicb.2025.1535002)
Supplement: Supplementary file 1 [file Table_1.docx]

Supplemental Table 1. All hit drugs with a selective index greater than 10

| Compound Number | Targeted Pathway | CC50 | EC50 | | | SI | | |
| --- | --- | --- | --- | --- | --- | --- | --- | --- |
|  |  |  | IL-8 | IP-10 | MCP-1 | IL-8 | IP-10 | MCP-1 |
| GNF-7 | Protein Tyrosine Kinase | 33.00 | <0.005 | 0.02 | 0.01 | 6600 | 1650 | 3300 |
| GZD824 | Protein Tyrosine Kinase | 4.80 | <0.005 | <0.005 | <0.005 | 960 | 960 | 960 |
| Bafetinib (INNO-406) | Protein Tyrosine Kinase | 100.00 | 0.59 | 0.41 | 0.86 | 169 | 244 | 116 |
| Ponatinib (AP24534) | Protein Tyrosine Kinase | 1.50 | <0.005 | 0.06 | <0.005 | 300 | 25 | 300 |
| CNX-774 | Protein Tyrosine Kinase | 15.50 | 0.01 | 1.46 | 0.16 | 1550 | 11 | 97 |
| Regorafenib (BAY 73-4506) | Protein Tyrosine Kinase | 20.00 | 0.61 | 1.80 | 1.71 | 33 | 11 | 12 |
| CL-387785 (EKI-785) | Protein Tyrosine Kinase | 7.09 | 0.42 | 0.41 | 0.64 | 17 | 17 | 11 |
| Poziotinib (HM781-36B) | Protein Tyrosine Kinase | 24.13 | 0.34 | 0.57 | 0.66 | 71 | 42 | 37 |
| Tyrphostin 9 | Protein Tyrosine Kinase | 8.26 | 0.04 | 0.09 | 0.08 | 207 | 92 | 103 |
| Pelitinib (EKB-569) | Protein Tyrosine Kinase | 1.70 | 0.02 | 0.13 | 0.02 | 85 | 13 | 85 |
| WZ3146 | Protein Tyrosine Kinase | 14.00 | 0.01 | 0.68 | <0.005 | 1400 | 21 | 2800 |
| SC1 | MAPK | ＞100 | <0.005 | 0.01 | 0.04 | 20000 | 10000 | 2500 |
| FIIN-2 | Protein Tyrosine Kinase | 16.66 | 0.03 | 0.32 | 0.14 | 555 | 52 | 119 |
| LY2874455 | Protein Tyrosine Kinase | 3.40 | <0.005 | <0.005 | <0.005 | 680 | 680 | 680 |
| IKK-16 (IKK Inhibitor VII) | NF-κB | 8.90 | <0.005 | <0.005 | <0.005 | 1780 | 1780 | 1780 |
| IMD 0354 | NF-κB | 1.10 | 0.02 | 0.08 | 0.06 | 55 | 14 | 18 |
| TPCA-1 | NF-κB | 11.00 | 0.28 | 0.07 | 0.12 | 39 | 157 | 92 |
| Oclacitinib | Protein Tyrosine Kinase | 50.00 | 0.05 | 0.18 | 0.19 | 1000 | 278 | 263 |
| Peficitinb (ASP015K, JNJ-54781532) | Protein Tyrosine Kinase | 34.70 | 0.01 | 0.02 | 0.01 | 3470 | 1735 | 3470 |
| Cerdulatinib (PRT062070, PRT2070) | Protein Tyrosine Kinase | 3.50 | <0.005 | 0.05 | 0.02 | 700 | 70 | 175 |
| Decernotinib (VX-509) | Protein Tyrosine Kinase | 50.00 | 0.02 | 0.10 | 0.05 | 2500 | 500 | 1000 |
| XL019 | Protein Tyrosine Kinase | 26.70 | 0.51 | 0.99 | 0.94 | 52 | 27 | 28 |
| Tofacitinib (CP-690550) Citrate | Protein Tyrosine Kinase | ＞100 | 0.02 | 0.04 | 0.04 | 5000 | 2500 | 2500 |
| S-Ruxolitinib (INCB018424) | Protein Tyrosine Kinase | 100.00 | 0.03 | 0.08 | 0.06 | 3333 | 1250 | 1667 |
| Baricitinib (LY3009104, INCB028050) | Protein Tyrosine Kinase | >100 | 0.07 | 0.04 | 0.08 | 1429 | 2500 | 1250 |
| CEP-33779 | Protein Tyrosine Kinase | 10.50 | 0.36 | 0.22 | 0.24 | 29 | 48 | 44 |
| Tofacitinib (CP-690550,Tasocitinib) | Protein Tyrosine Kinase | >100 | 0.01 | 0.02 | 0.05 | 10000 | 5000 | 2000 |
| CYT387 | Protein Tyrosine Kinase | 5.40 | 0.30 | 0.48 | 0.03 | 18 | 11 | 180 |
| AZ 960 | Protein Tyrosine Kinase | 6.00 | <0.005 | 0.01 | <0.005 | 1200 | 600 | 1200 |
| LY2784544 | Protein Tyrosine Kinase | 26.00 | <0.005 | 0.01 | <0.005 | 5200 | 2600 | 5200 |
| AZD1480 | Protein Tyrosine Kinase | 13.46 | 0.11 | 0.10 | 0.03 | 122 | 135 | 449 |
| Ruxolitinib (INCB018424) | Protein Tyrosine Kinase | 100.00 | 0.12 | 0.07 | 0.03 | 833 | 1429 | 3333 |
| TAK-733 | MAPK | 45.00 | <0.005 | 1.40 | 0.04 | 9000 | 32 | 1125 |
| PD0325901 | MAPK | >100 | <0.005 | 0.69 | 0.10 | 20000 | 145 | 1000 |
| INK 128 (MLN0128) | PI3K/Akt/mTOR | 5.90 | 0.04 | 0.12 | 0.04 | 148 | 49 | 148 |
| LY2228820 | MAPK | 50.00 | <0.005 | 3.70 | 2.23 | 10000 | 14 | 22 |
| BX-912 | PI3K/Akt/mTOR | >100 | 0.41 | 1.05 | 0.36 | 244 | 95 | 278 |
| AZ 628 | MAPK | >100 | 0.10 | 0.97 | 0.18 | 1000 | 103 | 556 |
| Dasatinib | Protein Tyrosine Kinase | >100 | 2.06 | 1.30 | 0.14 | 49 | 77 | 714 |
| Fostamatinib (R788) | Protein Tyrosine Kinase | 40.00 | 0.34 | 0.50 | 0.17 | 118 | 80 | 235 |
| R788 (Fostamatinib) Disodium | Protein Tyrosine Kinase | 7.40 | 0.06 | 0.39 | 0.16 | 123 | 19 | 46 |
| R406 (free base) | Protein Tyrosine Kinase | >100 | 2.75 | 0.70 | 1.13 | 36 | 143 | 88 |
| R406 | Protein Tyrosine Kinase | >100 | 0.82 | 3.50 | 0.27 | 122 | 29 | 370 |
| SGI-7079 | Protein Tyrosine Kinase | 3.85 | <0.005 | <0.005 | <0.005 | 770 | 770 | 770 |
| Sunitinib Malate | Protein Tyrosine Kinase | 24.86 | 0.05 | 0.66 | 0.35 | 497 | 38 | 71 |
| BIRB 796 (Doramapimod) | MAPK | >100 | 5.22 | 9.20 | 10.00 | 19 | 11 | 10 |
| Ibrutinib (PCI-32765) | Protein Tyrosine Kinase | >100 | 1.22 | 9.90 | 0.30 | 82 | 10 | 333 |
